# Tuberculosis preventive treatment uptake among people living with HIV during COVID-19 period in Addis Ababa, Ethiopia: a retrospective data review

**DOI:** 10.1186/s12879-024-09403-z

**Published:** 2024-05-17

**Authors:** Senedu Bekele Gebreegziabher, Akililu Alemu Ashuro, Tsegaye Hailu Kumssa, Melese Yeshambaw Teferi, Endawoke Amsalu Alemayue, Daniel Gemechu Datiko, Solomon Abebe Yimer, Mulatu Biru Shagre

**Affiliations:** 1 Armauer Hansen Research Institute (AHRI), Addis Ababa, Ethiopia; 2 USAID Eliminate TB Project Health Programs Group Management Sciences for Health, Addis Ababa, Ethiopia; 3 Vaccine Research and Development Department, Coalition for Epidemic Preparedness Innovations (CEPI), Oslo, Norway; 4 Faculty of Medicine, Unit for Genome Dynamics, University of Oslo, Oslo, Norway; 5 USAID Eliminate TB Project KNCV Tuberculosis Foundation-Ethiopia, Addis Ababa, Ethiopia

**Keywords:** Tuberculosis preventive treatment, COVID-19, Uptake status, People living with HIV, Addis Ababa

## Abstract

**Background:**

Screening for tuberculosis (TB) and providing TB preventive treatment (TPT) along with antiretroviral therapy is key components of human immune deficiency virus (HIV) care. The uptake of TPT during the coronavirus disease 2019 (COVID-19) period has not been adequately assessed in Addis Ababa City Administration. This study aimed at assessing TPT uptake status among People living with HIV (PLHIV) newly initiated on antiretroviral therapy during the COVID-19 period at all public hospitals of Addis Ababa City Administration, Ethiopia.

**Methods:**

A retrospective data review was conducted from April-July 2022. Routine District Health Information System 2 database was reviewed for the period from April 2020-March 2022. Proportion and mean with standard deviation were computed. Logistic regression analysis was conducted to assess factors associated with TPT completion. A *p*-value of < 0.05 was considered statistically significant.

**Results:**

A total of 1,069 PLHIV, aged 18 years and above were newly initiated on antiretroviral therapy, and of these 1,059 (99.1%) underwent screening for TB symptoms. Nine hundred twelve (86.1%) were negative for TB symptoms. Overall, 78.8% (719) of cases who were negative for TB symptoms were initiated on TPT, and of these 70.5% and 22.8% were completed and discontinued TPT, respectively. Of 719 cases who were initiated on TPT, 334 (46.5%) and 385 (53.5%) were initiated on isoniazid plus rifapentine weekly for three months and Isoniazid preventive therapy daily for six months, respectively. PLHIV who were initiated on isoniazid plus rifapentine weekly for three months were more likely to complete TPT (adjusted odds ratio [AOR],1.68; 95% confidence interval [CI], 1.01, 2.79) compared to those who were initiated on Isoniazid preventive therapy daily for six months.

**Conclusion:**

While the proportion of PLHIV screened for TB was high, TPT uptake was low and far below the national target of achieving 90% TPT coverage. Overall a considerable proportion of cases discontinued TPT in this study. Further strengthening of the programmatic management of latent TB infection among PLHIV is needed. Therefore, efforts should be made by the Addis Ababa City Administration Health Bureau authorities and program managers to strengthen the initiation and completion of TPT among PLHIV in public hospitals.

## Background

A quarter of the world’s population is infected with *Mycobacterium tuberculosis* (Mtb), a bacterium that causes TB [1]. Most people infected with Mtb are asymptomatic and classified as having latent TB infection (LTBI), which is the presence of immune responses to previously acquired Mtb infection without clinical evidence of active TB [2, 3].

About 5–10% of people with LTBI can develop active TB disease during their life time [4]. The risk of progressing from latent to active TB, however, can be higher among people with compromised immunity due to such as HIV infection, therapy that suppresses immunity, and among young age group [5]. For instance, the risk of developing active TB is about 15–22 times higher among PLHIV compared to people without HIV [2, 6], and the annual risk of developing active TB among individuals with untreated HIV is 3–16%, which is nearly the life time risk of TB among the general population. PLHIV who develop TB have also a high risk of mortality compared to people with TB alone [2].

The World Health Organization (WHO) has developed a guideline for the programmatic management of LTBI in PLHIV to prevent TB diseases. The guideline states that adults and adolescents living with HIV should be screened for TB according to a clinical algorithm, and for those who do not report any of the symptoms of TB, including current cough, fever, weight loss or night sweats, TPT should be offered, regardless of antiretroviral treatment (ART) status [3]. Screening for TB and treating LTBI along with the commencement of ART is the key components of HIV care [2].These interventions are imperative to reduce TB incidence among PLHIV [7].

TPT consists of a course of one or more anti-tuberculous drugs to treat persons with LTBI who are at high risk of progressing to active TB disease [5]. Preventive treatment reduces the risk of progression to active TB disease for individuals with LTBI, and also averts future Mtb transmission in the communities [8]. TPT is effective and safe for treatment of LTBI among PLHIV [9]. The efficacy of currently available TPT ranges from 60 to 90% [3]. Although TPT is essential and cost-effective component of HIV care, and has been recommended as a standard of care for over a decade, it has remained highly underutilized [10].

Ethiopia is one of the high TB and TB/HIV burden countries in the world [1]. The country notified a total of 104, 606 new TB cases in 2021. Eighty two percent of the total new TB cases knew their HIV status, of whom 5.2% were co-infected with HIV [11]. Considering the higher risk of progression to active TB and the deadly consequence of developing the disease, Ethiopia has been implementing Isoniazid preventive therapy (IPT) provision for PLHIV since 2007 [12]. The country adopted the WHO updated and consolidated guidelines to access shorter and safer TPT, and since 2020, the country has been implementing isoniazid plus rifapentine (HP) regimen provision for PLHIV [13]. Furthermore, Ethiopia has continued its commitment to achieve the United Nations High-Level Meeting (UNHLM) TPT target [14]. Accordingly, eligible individuals for TPT are being identified based on clinical symptom-based TB screening (clinical algorithm). The current TPT options that Ethiopia uses in the programmatic management of LTBI for PLHIV include isoniazid plus rifapentine (3HP) weekly for three months and daily IPT for Six months [13, 14]. In Ethiopia, uptake of TPT among PLHIV was 47% in 2020. This achievement was lower compared to the national target of achieving 90% TPT uptake for the year [15]. In 2021, TPT uptake among PLHIV increased to 64% at national level [16]. However, although there has been a progress, the achievement shortfalls compared to the national target the country has set to achieve.

Evidence showed a detrimental effect of COVID-19 pandemic on TB prevention and control activities [17, 18]. Ethiopia reported the first confirmed COVID-19 case on 13th March 2020 [19]. TB prevention and control services are part of essential health services in Ethiopia [20], and the Ministry of Health-Ethiopia set a guide for maintaining essential health services during the COVID-19 pandemic [21]. In Ethiopia, some studies assessed the impact of COVID-19 pandemic on TB control activities [22–24]. A study conducted in Addis Ababa reported a decline of 44.7% IPT uptake among children during the COVID-19 period [25]. In a pre-COVID-19 era, reports of few studies from Northeast Ethiopia, Northern-Ethiopia, and Southwest Ethiopia showed IPT uptake of 55%, 62%, and 66.5% among PLHIV, respectively [26–28]. A former study conducted at public health facilities in Addis Ababa showed that 28.7% of PLHIV had been treated with IPT [29]. Nonetheless, the uptake status of TPT among PLHIV during the COVID-19 period has not been adequately assessed generally in Ethiopia and particularly in Addis Ababa; the capital city of Ethiopia comprised more than 50% of total confirmed COVID-19 cases within the country [30]. Adequate information about LTBI management among PLHIV is important to identify potential areas of improvement and suggest strategies that will improve program performance. Thus, this study aimed at assessing the TPT uptake status among PLHIV who were newly initiated on ART during the first two years of the COVID-19 period (April 2020 to March 2022) at public hospitals of Addis Ababa City Administration, Ethiopia.

## Methods

### Study design and setting

Health facility based retrospective data review was conducted at public hospitals of Addis Ababa City Administration to assess the level of TPT uptake among PLHIV newly initiated on ART during COVID-19 period. Addis Ababa is the capital city of Ethiopia, with an estimated population of over 4.7 million in 2021 [31]. Six government hospitals and 100 public health centers were providing health services including TB and HIV health services (*HIV* testing, *prevention, treatment and care services*) in the city during the study period [32]. At health facilities, TB screening for adults living with HIV is being performed using a clinical algorithm. Accordingly, for those who do not report any one symptoms suggestive of active TB disease, including current cough, any fever, unintentional weight loss, and any night sweats, TPT should be initiated regardless of CD4 count and ART status. However, for those cases who report any one of the TB symptoms, further investigations to diagnose TB and other diseases are recommended [13, 14]. Addis Ababa City Administration Health Bureau is responsible for the health-care administration in the city.

### Data sources, data collection and management

We retrospectively reviewed the Routine District Health Information System 2 (DHIS 2) ART service database at all public hospitals of Addis Ababa City Administration to assess the level of TPT uptake among adults PLHIV, aged 18 years and above who were newly initiated on ART for the period starting from April 2020 to March 2022 (the first two years of the COVID-19 period) and who were screened negative for TB symptoms. PLHIV, aged less than 18 years who were newly initiated on ART during the same period were excluded. A structured data capturing template tailored from DHIS 2 was prepared and used to collect data related to TB screening and TPT uptake. Four trained data collectors reviewed and collected the data from April to July 2022. The data collected included the demographic and clinical characteristics of the cases (Age, sex, functional status, WHO clinical stage, TB screening status, TB screening result, and TPT status). The overall activities and entire process of data collection were led by investigators.

### Data analysis

The collected routine DHIS 2 data were cleaned, checked for correctness and consistency, and finally entered into a Redcap database, and the cleaned data were exported into Stata version 17 statistical software packages for statistical analysis. Descriptive statistics such as frequency, proportion, and mean with standard deviation (SD) were computed. To assess changes in TB prevention service performance, we compared the achievement for each of the selected service performance indicators, including number of cases who were newly initiated on ART, number of cases who were screened for TB symptoms, number of cases screened negative for TB symptoms, number of cases who were screened negative for TB symptoms and started on TPT, number of cases who completed TPT, number of cases who discontinued TPT, and number of cases who were screened negative for TB symptoms but not started on TPT between the second year (April 2021-March 2022) and the first year (April 2020-March 2021) on a quarterly basis during the COVID-19 period. We used the reported frequency of each quarter in the first year during COVID-19 period as a baseline to compare with reported frequency of each quarter (the corresponding quarter) in the second year during the COVID-19 period. The relative percentage changes of the services performance between corresponding quarters across the two years period were computed. Logistic regression analysis was conducted to assess the factors associated with completion of TPT. Variables with *p*-values of < 0.25 in the bivariate analysis were included in multivariate analysis. Crude odds ratio (COR) and adjusted odds ratio (AOR) with 95% confidence interval (CI) were used to assess the strengths of the association between variables and TPT completion. A *p*-value of < 0.05 was considered statistically significant.

### Ethical consideration

The Armauer Hansen Research Institute (AHRI)/All Africa Leprosy Rehabilitation and Training Center (ALERT) ethics review committee approved this study and gave waver of informed consent (PO/07/22). In addition, permission to review the required routine DHIS 2 ART service database was obtained from each hospital authority. The reviewed data used in this study were collected anonymously.

## Results

The routine DHIS 2 reports of 1,069 PLHIV who were newly initiated on ART during the period from April 2020 to March 2022 at six public hospitals of Addis Ababa City Administration were reviewed. Of these 56.5% were female and 42.8% were male. The mean age of the cases was 38.5 years (SD: ±11.2 years), and 34.9% of the cases were categorized in the age group 35–44 years. About 75% of cases had working functional status, and 56% were classified in WHO clinical stage T1 (Table 1).

**Table 1:** Demographic and clinical characteristics of PLHIV, April 2020 to March 2022, Addis Ababa, Ethiopia. *N* = 1069

Variables	Frequency	Percent (%)
Age group (years)		
18–24	98	9.2
25–34	292	27.3
35–44	373	34.9
45–54	203	18.9
55–64	77	7.2
65^+^	24	2.2
Gender		
Male	457	42.8
Female	604	56.5
Functional status		
Ambulatory	122	11.4
Bed ridden	62	5.8
Working	802	75.0
WHO clinical stage		
T1	599	56.0
T2	80	7.5
T3	101	9.5
T4	132	12.4

This table shows the demographic and clinical characteristics of PLHIV who were newly initiated on ART from April 2020 to March 2022 (COVID-19 period), at public hospitals of Addis Ababa City Administration, Ethiopia

Note: For about 2 (0.2%), 8 (0.7%), 83 (7.8%), and 157 (14.6%) of the cases age, type of gender, functional status, and the WHO clinical stage were not recorded, respectively

### TPT among PLHIV newly initiated on ART during COVID-19 period

Of 1,069 PLHIV who were newly initiated on ART in the period April 2020-March 2022, 99.1% were screened for TB symptoms, and of these 912 (86.1%) were negative for TB symptoms. Of 912 cases who were screened negative for TB symptoms, 78.8% were initiated on TPT and 21.2% were not initiated treatment during the period. Of those who started TPT, 70.5% completed treatment, 22.8% discontinued TPT, and 6.7% of cases were taking TPT. Of the total 719 cases who were initiated on TPT, 46.5% and 53.5% were started on 3HP and IPT, respectively. Of 334 cases who started 3HP, 76.6% completed treatment, 16.5% discontinued, and 6.9% were taking their treatment. Of 385 cases who were initiated on IPT, 65.2% completed treatment, 28.3% discontinued, and 6.5% cases were taking IPT during the period (Table 2).

**Table 2:** TPT uptake among PLHIV, Addis Ababa, Ethiopia, April 2020 to March 2022, *N* = 1069

Variables	Frequency	Percent (%)
TB symptoms screening status		
Screened for TB	1,059	99.1
Not recorded	10	0.9
TB symptoms screening results		
Positive	147	13.9
Negative	912	86.1
TPT initiation		
Started TPT	719	78.8
Not started TPT	193	21.2
TPT treatment outcome		
Completed TPT	507	70.5
Discontinued TPT	164	22.8
Currently on TPT	48	6.7
TPT initiation by regimen		
Started 3HP	334	46.5%
Started IPT	385	53.5%
TPT treatment outcome by regimen		
Completed 3HP	256	76.6%
Completed IPT	251	65.2%
Discontinued 3HP	55	16.5%
Discontinued IPT	109	28.3%
Currently on 3HP	23	6.9%
Currently on IPT	25	6.5%

This table shows TPT uptake among PLHIV newly initiated on ART at public hospitals, Addis Ababa City Administration, Ethiopia, April 2020 to March 2022

### Trends in TPT across quarters during COVID-19 period

Program performance indicators trend analysis across quarters for the period April 2021-March 2022 compared to April 2020-March 2021 showed that ART initiation increased by 15.4% in April-June 2021,1.6% in July-September 2021, 1.4% in October-December 2021, and 5.3% in January-March 2022 compared to the same quarters performance during the first year of COVID-19 period. Similarly, TB screening increased by 19.6% in April-June 2021, 2.4% in July-September 2021, and 4.5% in January-March 2022 compared to the same quarters performance in the previous year. In April-June 2021 and in July-September 2021, TPT uptake increased by 28.8% and 2.2%, respectively compared to the same period performance in the first year. For the period October-December 2021, TPT uptake reduced by 2% compared to in October-December 2020 performance. In the periods April-June 2021 and July-September 2021, TPT completion increased by 38.9% and 7.1%, respectively compared to the same quarters performance in the first year during COVID-19 period. TPT completion continuously decreased by 12.3% in October–December 2021 and 58.8% in January-March 2022 compared to the same quarters performance during the previous year. In the period July-September 2021 the number of cases discontinued TPT decreased by 22.2% compared to the number in July-September 2020. The number of cases discontinued TPT increased by 8.3% in October-December 2021 and 28.6% in January-March 2022 compared to the periods October to December 2020 and January -March 2021 number. Across quarters for the periods April- December 2021, the numbers of PLHIV who were screened negative for TB symptoms, but not initiated on TPT reduced by 13% in April-June 2021, 24.1% in July-September, and 11.1% in October-December 2021 compared to the same quarters number in the first year during COVID-19 period. In the last quarter of the study period (January-March 2022), the number of PLHIV who were not initiated on TPT increased by 18.2% compared to the mean number for the period in January-March 2021 (Table 3).

**Table 3:** Comparison of service performance indicators across quarters from April 2020-March 2022, Addis Ababa, Ethiopia

Service performance indicators	Number of cases across quarters for the period April 2020-March 2022											
	Apr- Jun 2020	Apr-Jun 2021	^¶^Percentage change	Jul-Sep 2020	Jul- Sep 2021	Percentage change	Oct-Dec 2020	Oct- Dec 2021	Percentage change	Jan-Mar 2021	Jan-Mar 2022	Percentage change
^╪^ Cases newly initiated on ART	117	135	+ 15.4%	127	129	+ 1.6%	144	146	+ 1.4%	132	139	+ 5.3%
Cases screened for TB symptoms	112	134	+ 19.6%	126	129	+ 2.4%	144	144	0.0%	132	138	+ 4.5.%
Cases screened negative for TB symptoms	96	114	+ 18.8%	118	114	-3.4%	125	119	-4.8%	111	115	+ 3.6%
Cases screened negative for TB symptoms and initiated on TPT	73	94	+ 28.8%	89	91	+ 2.2%	98	96	-2.1%	89	89	0.0%
Cases completed TPT	54	75	+ 38.9%	70	75	+ 7.2%	73	64	-12.3%	68	28	-58.8%
Cases discontinued TPT	17	17	0.0%	18	14	-22.2%	24	26	+ 8.3%	21	27	+ 28.6%
Cases screened negative for TB symptoms but not initiated on TPT	23	20	-13.04%	29	22	-24.13%	27	24	-11.1%	22	26	+ 18.2%

This table shows the TB prevention service performance across quarters for the period April 2021-March 2022 (the second year during COVID-19) compared to the corresponding baseline quarters for the period April 2020 – March 2021 (the first year during COVID-19) performance at public hospitals, Addis Ababa City Administration, Ethiopia^╪^ cases: PLHIV who were newly initiated on ART. ^¶^ Percentage change: Percentage change in the number in the two quarters

### TPT among PLHIV newly initiated on ART across the six hospitals

The proportion of PLHIVwho were screened for TB and initiated on TPT ranged from 94.6 − 99.7% and 76.2 − 82% across the hospitals in the period April 2020-March 2022. Of all cases screened negative for TB symptoms, 22.9%, 23.8%, 23.5%, and 22.9% were not initiated on TPT at Menelik Hospital, Ras Desta Hospital, Gandhi Hospital, and Yekatit 12 Hospital, respectively (Table 4).

**Table 4:** TPT uptake disaggregated by public hospitals, April 2020 to March 2022, Addis Ababa, Ethiopia. *N* = 1069

Health facilities	Started ART ^*^ *N*	^¶^Screened for TB symptoms *N* (%)	^φ^Screened negative for TB symptoms *N* (%)	^ϕ^Initiated TPT *N* (%)	$Not initiated TPT *N* (%)
Zewditu Hospital	326	325 (99.7%)	270 (83.1%)	219 (81.1%)	51 (18.9%)
Trunesh Beijing Hospital	152	151 (99.3%)	128 (84.8%)	105 (82.0%)	23 (18.0%)
Yekatit 12 Hospital	196	195 (99.5%)	166 (85.1%)	128 (77.1%)	38 (22.9%)
Gandhi Hospital	37	35 (94.6%)	34 (97.1%)	26 (76.5%)	8 (23.5%)
Menelik Hospital	218	215 (98.6%)	192 (89.3%)	148 (77.1%)	44 (22.9%)
Ras Desta Hospital	140	138 (98.6%)	122 (88.4%)	93 (76.2%)	29 (23.8%)
Total	1,069	1,059 (99.1%)	912 (86.1%)	719 (78.8%)	193 (21.2%)

This table shows TPT uptake among PLHIV newly initiated on ART from April 2020 to March 2022, disaggregated by public hospitals, Addis Ababa City Administration, Ethiopia

N: number; ^¶^ Screened for TB: screened for TB using symptom algorism; ^φ^TB screening negative: negative TB screening result;^ϕ^Started TPT: screened negative for TB symptoms and started TPT;^$^ not started TPT: screened negative for TB symptoms, but not started TPT

**Fig. 1:**
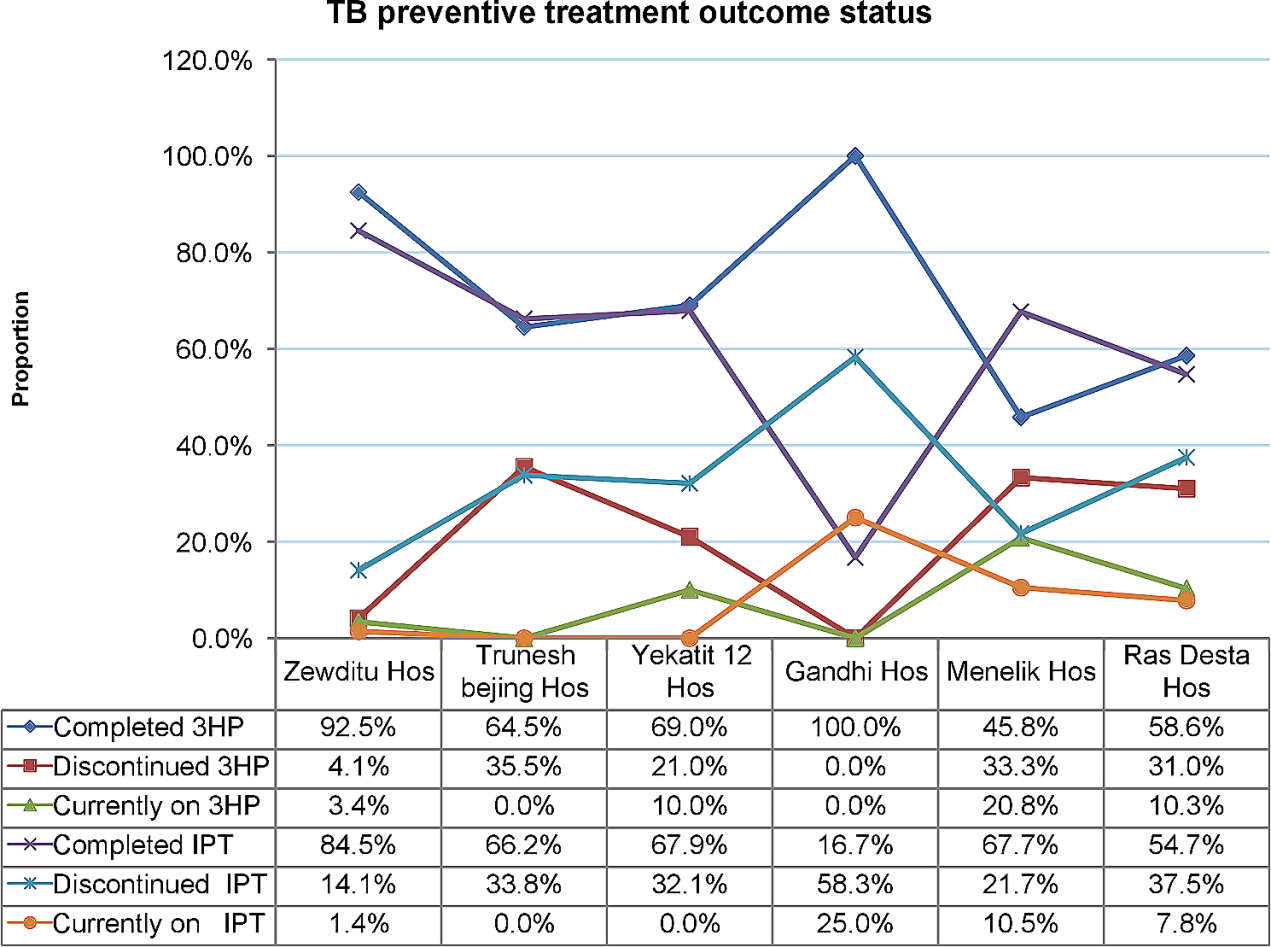
TPT outcome status, April 2020-March 2022, disaggregated by treatment regimens, Addis Ababa, Ethiopia This graph illustrates the TPT outcome status of PLHIV newly initiated on ART in the period April 2020-March 2022, disaggregated by treatment regimens, across public hospitals, Addis Ababa City Administration, Ethiopia

Of the 334 cases who were initiated 3HP regimen, 148 (44.3%), 31 (9.3%), 100 (29.9%), 2 (0.5%), 24 (7%), and 29 (9%) cases started at Zewditu Hospital, Trunesh Beijing Hospital, Yekatit 12 Hospital, Gandhi Hospital, Menelik Hospital, and Ras Desta Hospital, respectively. A total of 385 cases including 71 (18.4%) at Zewditu Hospital, 74 (19.2%) at Trunesh Beijing Hospital, 28 (7.3%) at Yekatit 12 Hospital, 24 (6.2%) at Gandhi Hospital, 124 (32.2%) at Menelik Hospital, and 64 (16.6%) at Ras Desta Hospital were initiated on IPT. Of those who were initiated 3HP and IPT at Zewditu Hospital, 92.5% and 84.5% of cases completed treatment, respectively. The proportion of cases who completed 3HP regimen at Menelik Hospital and Ras Desta Hospital was 45.8% and 58.6%, respectively. 17% of the cases at Gandi Hospital and 54.7% at Ras Desta Hospital completed IPT. 36% of cases at Trunesh Beijing Hospital, 33.3% at Menelik Hospital, and 31.0% at Ras Desta Hospital discontinued 3HP. 58% of cases discontinued IPT at Gandi Hospital during the period April 2020-March 2022 (Fig. 1).

### Factors associated with TPT completion

In multivariate analysis PLHIV who were initiated on TPT at Gandhi Hospital, Trunesh Beijing Hospital, Yekatit 12 Hospital, Menelik Hospital, and Ras Desta Hospital had 95% (AOR, 0.05; 95% CI, 0.01, 0.16), 85% (AOR, 0.15; 95% CI, 0.07, 0.34), 80% (AOR, 0.20; 95% CI, 0.09, 0.42),74% (AOR, 0.26; 95% CI, 0.12, 0.54, and 85% (AOR, 0.15; 95% CI, 0.07, 0.33), lower TPT completion, respectively compared to those who were initiated on TPT at Zewditu Hospital. PLHIV who were initiated 3HP regimen were more likely to complete (AOR, 1.68; 95% CI, 1.01, 2.79) compared to those who were initiated on IPT (Table 5).

**Table 5:** Factors associated with TPT completion, April 2020 to March 2022, Addis Ababa, Ethiopia

Variables	Number of cases who initiated TPT	Number of cases completed TPT	COR (95% CI)	*P*-value	AOR (95% CI)	*P*-value
**Gender**						
Female	427	299 (70.0%)	0.99 (0.69–1.42)	0.97	1.22 (0.79–1.90)	0.37
Male	288	205 (71.2%)	1		1	
**Age group (years)**						
18–24	67	42 (62.7%)	0.82 (0.23–2.92)	076	0.85 (0.18–4.07)	0.84
25–34	187	115 (61.49%)	0.63 (0.19–2.03)	0.44	0.78 (0.18–3.35)	0.73
35–44	261	192 (73.56%)	1.14 (0.35–3.68)	0.82	1.10 (0.26–4.71)	0.89
45–54	134	102 (76.12%)	1.55 (0.46–5.24)	0.49	1.83 (0.40–8.35)	0.43
55–64	53	44 (83.02%)	3.67 (0.79–16.86)	0.09	2.86 (0.49–16.85)	0.25
65+	17	12 (70.58%)	1		1	
**WHO clinical stage**						
T1	464	320 (68.9%)	0.89 (0.45–1.77)	0.75	0.94 ( 0.39–2.19)	0.88
T2	61	47 (77.0%)	1.35 (0.54–3.39)	0.53	0.71 (0.24–2.08)	0.53
T3	48	39 (81.3%)	1.76 (0.63–4.95)	0.28	1.70 (0.54–5.35)	0.36
T4	54	38 (70.4%)	1		1	
**TPT regimen**						
6 H	385	251 (65.2%)	1		1	
3HP	334	256 (76.6%)	1.98 (1.37–2.87**)**	< 0.001*	1.68 (1.01–2.79)	0.04*
**Functional status**						
Working	605	435 (71.9%)	1		1	
Ambulatory	55	34 (61.8%)	0.61 (0.33–1.13)	0.12	0.47 (0.22–1.03)	0.06
Bed ridden	17	10 (58.8%)	0.61 (0.21–1.82)	0.38	0.68 (0.17–2.78)	0.59
**Health facilities**						
Zewditu Hospital	219	197 (89.9%)	1		1	
Gandhi Hospital	26	6 (23.1%)	0.03 (0.01–0.10)	< 0.001*	0.05 (0.01–0.16)	< 0.001*
Trunesh Beijing Hospital	105	69 (65.7%)	0.16 (0.08–0.29)	< 0.001*	0.15 (0.07–0.34)	< 0.001*
Yekatit 12 Hospital	128	88 (68.8%)	0.24 (0.12–0.46)	< 0.001*	0.20 (0.09–0.42)	< 0.001*
Menelik Hospital	148	95 (64.2%)	0.22 (0.12–0.42)	< 0.001*	0.26 (0.12–0.54)	< 0.001*
Ras Desta Hospital	93	52 (55.9%)	0.13 (0.65 − 0.25)	< 0.001*	0.15 (0.07–0.33)	< 0.001*

This table shows the factors associated with TPT completion among PLHIV newly initiated on ART for the period April 2020 to March 2022, at public hospitals, Addis Ababa City Administration, Ethiopia

*statistically significant

## Discussion

The COVID-19 pandemic posed substantial challenges on routine health services [33]. In this study, we assessed TB screening, TPT uptake, and associated factors of TPT completion among PLHIV newly initiated on ART during the first two years of the COVID-19 period (April 2020-March 2022). We also compared service performance indicators a quarter by quarter bases between the two years.

We observed that ART initiation, TB screening, TPT uptake, and TPT completion increased in April-June 2021 (the first quarter during the second year of the COVID-19 period) compared to in April-June 2020 (the first quarter during the first year of the COVID-19 period). Studies from various regions of Ethiopia showed reduction in routine health services performance including TB prevention and control during the early period of the COVID-19 pandemic [25, 34, 35]. This may be related to that April-June 2020 was the period when national lockdown declared to control COVID-19 in Ethiopia [36]. The country adopted a variety of response measures such as travel restrictions, reorganization of health facilities [25, 30], and reallocating human workforce and services towards responding to COVID-19 pandemic [30], which may hinder routine health services provision. Moreover, as part of response, there was intense media coverage about the COVID-19 outbreak, particularly during the early period of the pandemic, and this was a time when the daily COVID-19 reported cases in Ethiopia, particularly in Addis Ababa were high [30]. This might make the clients/patients fear of acquiring COVID-19 and may have forced them to stop using health care services.

Ruling out active TB proceeding to commencement of preventive treatment is one of the crucial steps in LTBI treatment pathway [3]. The national TB control program guideline of Ethiopia recommends clinical screening using symptom-based criteria as a requirement to identify those eligible for TPT and for initiating preventive treatment [13]. We observed increment in TB screening in April-June 2021 compared to in April-June 2020 (lockdown period). A study conducted in other region of Ethiopia showed a reduction trend in TB screening in routine HIV care services in the early period of COVID-19 pandemic [34]. The trend in TB screening also showed increment in the period July-September 2021 and January-March 2022 compared to July-September 2020 and January-March 2021 performance. Overall, 99% of cases were screened for TB symptoms during the study period, which might be explained by Addis Ababa comprised more than 50% of total confirmed COVID-19 cases within the country [30], and this condition may have forced health workers for performing simultaneous TB and COVID-19 screening for PLHIV during the pandemic period as both disease share same symptoms such as cough, fever, and shortness of breath [37, 38].

In this study, although increment trend was observed in TPT initiation in April to June 2021 and in July to September 2021 compared to in April to June 2020 (first quarter during lockdown period) and in July-September 2020 (second quarter during lockdown period), reduction was observed in October to December 2021compared to the same quarter performance in the first year. This may be partially related to decrement in the number of cases who were screened for TB and screened negative for TB symptoms during the same period as Table 2 showed. Overall, during the study period more than three-fourths of eligible cases were initiated on TPT. This is similar to the result of a former study from Eastern Ethiopia whereby 78.7% of PLHIV were initiated on TPT [39]. Nevertheless, the TPT uptake in this study was far below the national target of achieving 90% TPT coverage [15]. Intensive screening with TPT has the potential to significantly decrease the incidence of TB among high-risk groups like PLHIV and ultimately reduce transmission in the community [40].

Although a decreasing trend was observed in the number of cases discontinued TPT in the period July to September 2021 compared to in July to September 2020 (lockdown period), however, it continuously increased in October to December 2021 and in January to March 2022 compared to the same quarters number in the previous year during the COVID-19 period. We observed that overall, 22.8% of cases discontinued TPT during the study period. This is in line with a study conducted at health centers in Addis Ababa whereby 27.4% PLHIV who were on ART missed their appointments/visits for refill during the COVID-19 pandemic [41]. Our hospital-based analysis also showed that a substantial proportion of cases discontinued IPT at most of the hospitals, including Gandi, Yekatit 12, Trunesh Beijing, Menelik, and Ras Desta. Likewise, more than a third of cases in each of three hospitals, including Trunesh Beijing, Yekatit 12, and Ras Desta discontinued 3HP. The high proportion of cases discontinued TPT in the current study might be related to inadequate health education and psychosocial support for clients due to redirection of most health care providers to care COVID-19 cases [30]. In a recent meta-analysis, poor patient adherence, poor patient empowerment and proper counseling on IPT, fear of side effects and developing isoniazid resistant TB, and as well as lack of commitment of health managers to scale up the program were challenges in Ethiopia [42]. Discontinuation of TPT is an obstacle to effective TB control and has the potential of worsening the emergence of drug resistant TB and death [43]. Our result underscores the importance of strengthening adherence on TPT.

In this study, overall 22% of cases who were screened negative for TB symptoms were not initiated onTPT during the study period. A study from Brazil reported a reduction in TPT prescription during the COVID-pandemic [44]. In trend assessment, the number of PLHIV who were not started on TPT continuously declined across quarters in April to June 2021, in July to September 2021, and in October to December 2021 compared to the same quarters number in the first year during the pandemic period and this was found to be an encouraging trend; however, in the last quarter of the study period (January to March 2022) increment was observed. Similarly, our hospital based analysis revealed that nearly a quarter of PLHIV in each of four hospitals, including Yekatit12, Gandi, Menelik, and Ras Desta were not initiated on TPT. However, the reasons for not initiating TPT and discontinuing TPT after initiating were not documented in the data bases and ART registers. The possible explanation might be that the repurposing of health workers may have led to hesitation to provide routine services and continue recording the required information of each cases profile during the pandemic. PLHIV receiving ART remain at considerable risk for developing TB [29, 42]. TPT works synergistically with ART to reduce progression LTBI to active TB, mortality, and incidence in PLHIV [29, 45]. Reservoir of TB infection like PLHIV with the highest risk of progression to active TB must be addressed to end the TB epidemic globally [29].

More than half (70.5%) of cases completed TPT. Findings of a study from Brazil revealed 74% TPT completion during the COVID-19 pandemic [44]. In trend analysis, although TPT completion was increased in the first two quarters during the second year of the COVID-19 pandemic, however, decrement trend was observed in the periods October to December 2021 and January to March 2022. This may be related to increment of cases who discontinued TPT during the same quarters. Our study showed that cases who were initiated on 3HP were more likely to complete TPT than those who were initiated on IPT. In a recent systematic review and meta-analysis report, high completion rate of shorter, rifampicin-containing regimens including 3HP than isoniazid-based regimens has been reported [45]. The high 3HP completion in the current study may be due to the fact that 3HP regimen is simpler, requires fewer doses, and is taken for shorter period of time than IPT [46, 47]. In Ethiopia, the introduction of 3HP increased acceptance among health care providers [48]. The high 3HP completion in our study may also be related to the counseling service that health care providers have been providing and the commitment of PLHIV. In relation to concerns about toxicity and the long duration of treatment, acceptance and completion of IPT in PLHIV have been poor worldwide [49].

Cases who attended at Gandhi Hospital, Trunesh Beijing Hospital, Yekatit 12 Hospital, Menelik Hospital, and Ras Desta Hospital

were less likely to complete TPT than those who attended at Zewditu Hospital. This may be explained by Zewditu Hospital is a well-known model hospital which has a well-functioning HIV care program. The hospital is the first hospital started provision of ART services in Ethiopia in 2003 [50]. Zewditu Hospital has extensive experience in providing better HIV care services and a large number of PLHIV who had been enrolled for HIV care and put on ART [51]. This is also supported by the current data whereby near to a third (30.4%) of the cases were initiated on ART at this hospital.

The study attempts to assess the level of TPT uptake among PLHIV who were newly initiated on ART at all public hospitals of Addis Ababa City Administration during the COVID-19 period, and the results indicate important issues that need to be addressed to improve programmatic management of LTBI in the study hospitals. The study has potential limitations: Firstly, due to time and other resource constraints we had, the study was carried out only at government hospitals; therefore, the results do not reflect the TPT uptake status among PLHIV who attended at public health centers and private health facilities in Addis Ababa City Administration. Moreover, the study focused on adult PLHIV who were newly initiated on ART; hence the findings can not be generalized to all PLHIV who were newly enrolled into HIV care during the study period. Secondly, as the study used routine data, we could not report on the reasons for cases who were not initiated on TPT and who discontinued TPT because this information was not recorded in the data bases and ART registers. Thirdly, the study used routine data which were collected for programmatic purposes and patient management; therefore, some variables which might have an association with TPT completion were not routinely collected and recorded in the database, which is an inherent limitation of retrospective study designs.

## Conclusions

Most of the TB prevention service performance indicators showed increment during the second year of the COVID-19 period, which may indicate health service recovery. While the overall proportion of PLHIV who were screened for TB was high, TPT uptake was low and far below the national target of achieving 90% TPT coverage. Overall a considerable proportion of cases discontinued TPT and near to a quarter of cases who were screened negative for TB symptoms were not initiated on TPT during the period, which may have implications for LTBI progress to TB disease. Nonetheless, the reasons for not initiating TPT and discontinuation of TPT after initiation were not properly documented in the data sources. PLHIV who started TPT at Zewditu Hospital and cases who initiated 3HP regimen were predictors for TPT completion. Further strengthening of the programmatic management of LTBI for PLHIV is needed. Therefore, efforts should be made by the Addis Ababa City Administration Health Bureau authorities and program managers to strengthen TPT initiation and completion among PLHIV in public hospitals. Due emphasis should be given on recording the required information for all cases who are enrolled for HIV care. Additionally, digitalizing the documentation system is essential for improving the registration and tracking reasons for not initiating TPT and discontinuing treatment. These might enhance, monitor, and support evidence-informed decision-making. Additional research is needed to understand the reasons for the low TPT uptake and high discontinuation of TPT among PLHIV in the study hospitals.

## Data Availability

The datasets used and/or analyzed during the current study are available from the corresponding author on reasonable request.

## Abbreviations

AHRI: Armauer Hansen Research Institute
AOR: Adjusted odds ratio
ART: Antiretroviral therapy
CI: Confidence interval
CEPI: Coalition for Epidemic Preparedness Innovations
COVID-19: Coronavirus disease 2019
COR: Crude odds ratio
DHIS 2: District Health Information System 2
HIV: Human immune deficiency virus
HP: Isoniazid plus rifapentine
IPT: Isoniazid preventive therapy
LTBI: Latent TB infection
Mtb: Mycobacterium tuberculosis
MOH: Ministry of Health
PLHIV: People living with HIV
SD: Standard deviation
TB: Tuberculosis
TPT: TB preventive treatment
USAID: United States Agency For International Development
UNHLM: United Nations High-Level Meeting
WHO: World Health Organization
